# The influence of different pH on the electrophoretic behaviour of *Saccharomyces cerevisiae* modified by calcium ions

**DOI:** 10.1038/s41598-018-25024-4

**Published:** 2018-05-08

**Authors:** Agnieszka Rogowska, Paweł Pomastowski, Michał Złoch, Viorica Railean-Plugaru, Anna Król, Katarzyna Rafińska, Małgorzata Szultka-Młyńska, Bogusław Buszewski

**Affiliations:** 1 Centre for Modern Interdisciplinary Technologies Nicolaus Copernicus University, Wileńska 4, 87–100 Torun, Poland; 2 Department of Environmental Chemistry and Bioanalytics, Faculty of Chemistry, Nicolaus Copernicus University, Gagarina 7, 87-100 Torun, Poland

## Abstract

The effect of a different pH on *Saccharomyces cerevisiae* cells modified with calcium ions was investigated by the capillary zone electrophoresis technique. For the identification of the wild strain of *S*. *cerevisiae*, the ribosomal nucleic acid sequencing and internal transcribed spacer sequencing as well as spectrometric approach were applied. The potentiometric titration and Fourier transform infrared spectroscopy have shown the occurrence of active functional groups such as carboxyl, amine/hydroxyl, phosphate/hydrogen phosphate groups on the surface of native yeast cells. Moreover, the spectroscopy study in a medium infrared range was carried out to identify the functional groups of yeast cells that participate in calcium ions binding interaction. Furthermore, the microscopic and spectrometric analysis shows that the pH value of the calcium ions solution has a significant effect on the intensity yeast cells clumping. Additionally, the impact of yeast cell clumping on the electrophoretic behaviours was examined. The modification of surface functional groups by calcium ions significantly affected the efficiency of electrophoretic separation. However, these changes did not affect the accuracy of *S*. *cerevisiae* identification by MALDI equipment with BioTyper platform. These results form the analytical solution for coupling of electrophoresis and MALDI-TOF MS technique.

## Introduction

Capillary zone electrophoresis (CZE) is a well known and widely used technique enabling the separation, identification and electroanalysis of yeast or bacteria^1^. Despite such widespread use of CZE, this technique is in the process of evolution and requires the development of new methods for sample preparation. In recent years a lot of interest has been focused on the possibility of applying this technique to electroanalysis to provide the identification of wild stains of microorganisms in diagnostics laboratory. However, the electrophoretic analysis of such complicated systems as microorganisms can cause many difficulties associated with uncontrolled cell aggregation and adhesion to the inner surface of the capillary^2,3^. Over the years, several strategies aimimg at the elimination of the phenomenon of aggregation and adhesion, such as the addition of poly (ethylene oxide) (PEO) to buffer solution^1,4^ and inner capillary surface modification were proposed^5^. The new approach involves changes of functional groups on the microbial surface by divalent metal ions resulting in aggregation of cells controlled to a certain extent^6^. The most widely used thermodynamic model for the description of the aggregation phenomenon is DLVO (Derjaguin, Landau, Verwey, Overbeek) theory^2^. The physicochemical characterization of the microorganisms surface using instrumental analysis techniques is necessary to understand their behaviour during the electrophoretic analysis and to explain the mechanisms responsible for this process^1,2,4–6^.

One of the oldest eukaryotic microorganisms classified as fungi are yeasts. There are about 1500 species of yeasts and some of them, such as *Saccharomyces cerevisiae*, are used in brewing beer or baking^7^. Moreover, they are significant tools in molecular and cell biology – intensive studies concerning their genome or cell structure allowed to discover \e.g. many proteins involved in the cell cycle, signalling or metabolic pathways. *S*. *cerevisiae* can also be called a model organism because they are small with a short generation time and can be easily cultured^7^. The yeasts cell wall structure is composed of polysaccharides (glucans, chitin and mannans; about 85%) and proteins (about 15%)^8–10^. In glucans, glucose residues are bound through β-1,3 and β-1,6 bands, with other glucose molecules. Chitin is a linear polymer of β-1,4-linked N-acetylglucosamine (GlcNAc) and it forms microfibrils which are stabilized by hydrogen bonds. In mannans α-1,4- glycosidic bonds occur^11^. Among them there are, mannoproteins and cell-wall proteins (CWPs). Mannoproteins are linked to polysaccharide non-covalently and CWPs are branched by β-1,6 or β-1,3 linkages of glucans^12^. Moreover, such a construction of the yeast cell wall results in there being many functional groups on its surface such as phosphate, carboxyl and amino groups. Under appropriate conditions of pH (above pK_a_ value), these groups are deprotonated, which allows their interaction with positive charged metal ions which results in cells flocculation^13^. Many metal ions such as Mg^2+^, Mn^2+^, Zn^2+^, Cd^2+^ or Co^2+^ have been described as yeast cell aggregation inducers. However, calcium cations are known as the most effective in flocculation promotion^14,15^. It is caused by the chemistry of calcium ions, especially in contrast to D-block elements, which did not create the aggregates in pH greater than 7^14,15^. The hypothesis of calcium - binding suggests that calcium ions cause cells aggregation by binding mostly carboxyl groups present on the yeast surface. The second theory is related to the lectin-like mechanism. According to this theory, zymolectins on the yeast cells are binding specifically to mannose residues on the adjacent cell. In this conditions, calcium ions are necessary to provide zymolectins correct conformations^16^.

Once the yeast cell has a complex wall structure and is capable to induce flocculation, the *Saccharomyces cerevisiae* can be considered a model of (bio)colloids. The (bio)colloids are characterized by the complex surface topography and the heterogeneous distribution of the electric charge^6,17^. The term “(bio)colloids” comes directly from the term “colloid”. “(Bio)colloids” are considered as the biologically heterogeneous systems which are intermediate between the real solutions and suspensions. In (bio)colloids, the particles of the dispersed phase are called microns and their diameter ranges from 0.2 μm to 0.1 mm^17^. (Bio)colloids are classified into lyophobic - repulsive dispersion medium and lyophilic - showing affinity of the dispersed phase to the dissipation phase. On the surface of (bio)colloids, a double electric layer is formed. The first one is a layer, irreversibly related to the real surface of the (bio)colloid called a chemisorption layer. The second layer is formed by reversible physical adsorption. The kinetic stability of the (bio)colloid is determined by the existence of a surface charge - zeta potential^6,17^.

The aim of this study was to investigate the relationship between the heterogeneity and charge of the microorganism surface as well as behaviour of tested (bio)colloids during electrophoretic analysis. Furthermore, the impact of the cells surface modification by calcium ions on clumping of (bio)colloids and on the effectiveness of the electrophoretic mobility was examined. *Saccharomyces cerevisiae* was used as a model of (bio)colloids. The novel approach of the microbial sample preparation for the CZE analysis may constitute a significant contribution to the future use of this technique in diagnostics laboratory. Moreover the spectrometric analysis of yeast modified by calcium ions may be a foundation for the coupling of capillary electrophoresis and MALDI-TOF MS analysis to eliminate the preconcentration problem of microbiological samples.

## Results and Discussion

### Identification of Saccharomyces cerevisiae strain

**Figure 1:**
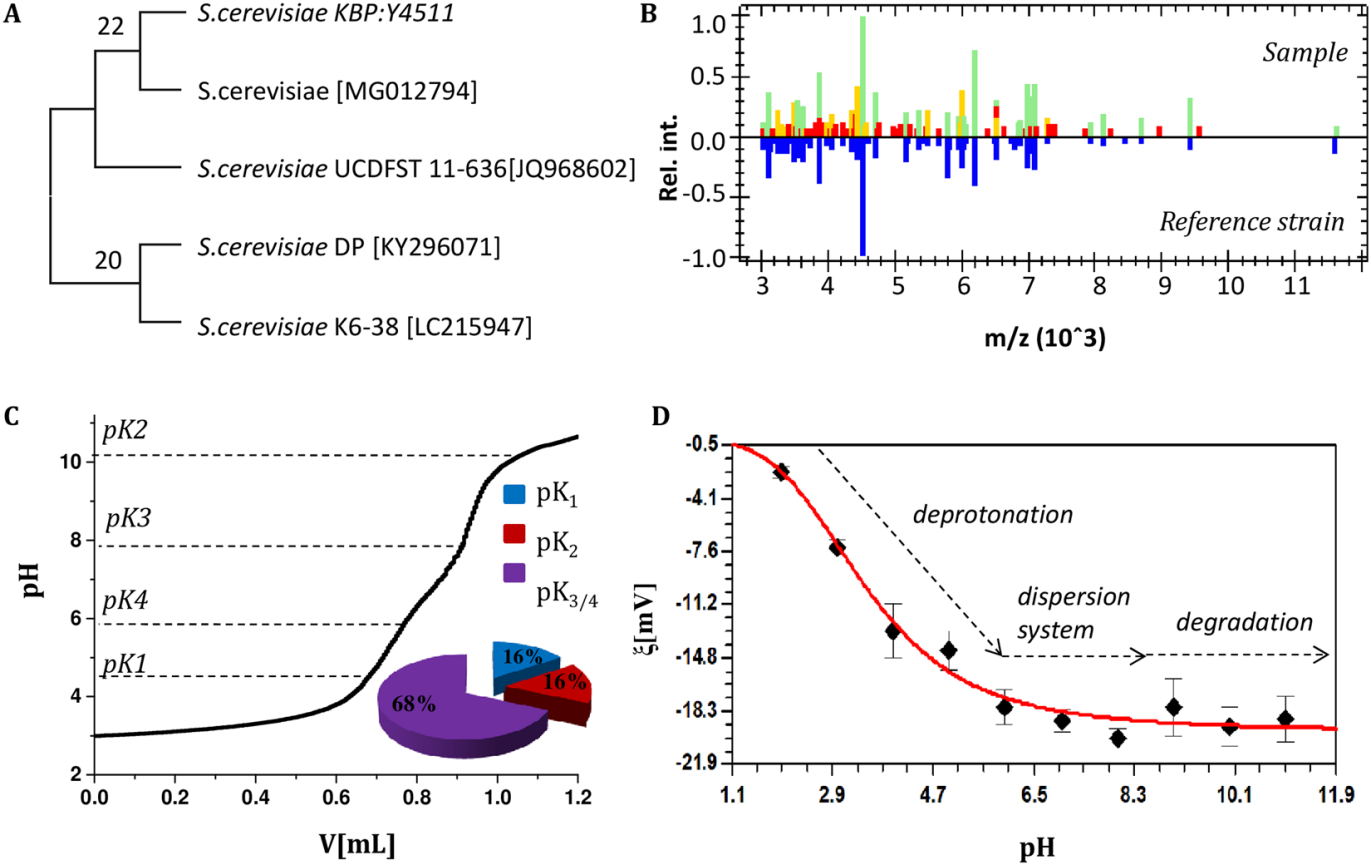
Phylogenetic tree of investigated *Saccharomyces cerevisiae* strain (**A**). MALDI-TOF MS spectra of native tested yeast and *Saccharomyces cerevisiae* INVSc1 BRL (**B**). Potentiometric titration curve of *S*. *cerevisiae* in anaerobic conditions and number of pK_a_ obtained for surface functional groups of *S*. *cerevisiae* (**C**). The zeta potential value of *S*. *cerevisiae* biocolloids in function of different pH (**D**).

The 28 S rDNA and ITS sequencing results revealed that the investigated yeast strain was identified as *Saccharomyces cerevisiae* [Accession number: MG012794] with 100% (539/539) identity and sequence overlap with the most similar *Saccharomyces cerevisiae* strain KBP:Y-4511 (28 S) or *Saccharomyces cerevisiae* strain Hitachi-SUY1 (ITS) **(**Fig. 1A**)**. Moreover, the molecular identification results were successfully confirmed by the analysis performed by the MALDI Biotyper platform at a species cutoff score value of 2.08 **(**Fig. 1B**)**. Classification and identification of the microorganisms are based on proteomic fingerprinting using the high-throughput matrix assisted laser desorption/ionization time-of-flight mass spectrometry. Specifically, the MALDI Biotyper CA System estimates highly abundant proteins which occur in every microorganism. The specific patterns of those proteins are applied to the credible and precise identification of a certain microbe by matching it against the FDA-cleared library. According to the manufacturer of the BioTyper software (Bruker Daltonics), scores of >2.0 allow for microorganisms identification at species level^18^.

### Characteristics of the surface properties of Saccharomyces cerevisiae

All of the yeast cell wall components such as polysaccharides, proteins, lipids and sterols are considered to constitute a system consisting of the core and the specific surface functional groups^9^. For polysaccharides, the core is a hexagonal carbon ring and the main surface groups are hydroxyl groups (−OH). In the case of phospho-manann, there are additional (di)hydrogen phosphate (H_2_PO_4_^−^/-HPO_4_^2−^) groups^19^. Each of them undergoes protonation and deprotonation reactions giving the electric charge on the cell surface.

In order to identify the surface functional groups of *S*. *cerevisiae*, potentiometric titration was performed. The analysis was carried out under anaerobic conditions due to the fact that yeasts conduct alcoholic fermentation resulting in the emission of carbon oxide(IV).

The pH range of these analysis ranged from 3 to 12. In order to confirm the viability of the test cells at such extremely low and high pH values, the microscopic analysis was carried out. Supplementary Table S1 shows the percentage of living and dead yeast cells at different pH and the Supplementary Fig. S2 shows the microscopic images of yeast at different pH. The results acquired indicate that in the entire pH range, yeast cells show quite good viability. Moreover, its cell wall is not damaged and cells are integrated.

Figure 1C shows the titration curve of the *S*. *cerevisiae*. The pK_a_ of yeast functional groups was calculated and were as follows: pK_1_ = 4.53 ± 0.43, pK_2_ = 10.24 ± 0.24, pK_3_ = 7.67 ± 0.38 and pK_4_ = 5.70 ± 0.59. The obtained pK_a_ values can be attributed to the protolytic equilibrium of the surface functional groups. The results show that the carboxyl (pK_a_ 2–6), amine/hydroxyl (pK_a_ 8.5–10.6) and phosphate/hydrogen phosphate (pK_a_ 5.5–7.2) groups have the dominant impact on the surface of *S*. *cerevisiae*^20^. Moreover, the quantitative share of particular functional groups on the surface of *S*. *cerevisiae* was investigated **(**Fig. 1C**)** - phosphate/hydrogen phosphate (H_2_PO_4_^−^/-HPO_4_^2−^) groups possess dominant (68%) quantitative participation which may indicate that the main component of the tested *S*. *cerevisiae* cells is phospho-mannan. It results from the molecular composition of yeast cells walls.

In order to determine the surface charge of *S*. *cerevisiae*, the zeta potential measurement was performed. Figure 1D shows the relationship between the zeta potential and the pH value of the yeast suspended in NaNO_3_ (5 mM) solution. The zeta potential value for the yeast in the pH at range 2–6 decreases from −3 to −18 mV, while for pH above 7 its value ranges from −19 to −20 mV. Our experimental data highlighted that with the increasing pH of the suspending solution, the absolute value of the zeta potential rises. Moreover, the zeta potential value greater than ±20 mV indicates the dispersion stability of the system^21^. This suggests that tested (bio)colloid is stable at pH higher than 8.

The knowledge of the main functional groups on the yeast cell surface and its pK_a_ values as well as of its dispersion stability allows the selection of sample preparation conditions for further analysis. Following the obtained results the pH for yeast cells modification should be equal or higher than 6, because in these conditions most of the functional groups begin to deprotonate and the system shows quite good stability of dispersion. However, the microscopic study indicates yeast cell incubation at pH higher than 9 and the change of the structure of the cells. Cytoplasm was significantly more vaculated than in cells in lower pH and its viability started to decrease **(**Supplementary Fig. S2**)**. For this reason pH 6, 8 and 9 were chosen to continue the study of cells modification by calcium ions.

### Characteristics of the cells surfaces properties after its modification with calcium ions

Ca^2+^ ions are considered to be one of the most important factors enabling aggregation of *S*. *cerevisiae* cells as a result of calcium-bridging of neighbouring cells by the binding surface-carboxyl groups^16^ or lectin-like mechanisms of flocculation in which Ca^2+^ enables the lectins to acquire a suitable active conformation and thus interact with carbohydrate residues of α-mannans present on the cell surface^22^. Moreover, regardless of cells aggregation mechanism, the pH value of culture medium may significantly affect the flocculation process since its changes alter the ionization of the functional residues present on the cell surface, mainly the carboxyl group, thus modifying the availability of Ca^2+ ^^23,24^. In order to confirm the surface modification of *S*. *cerevisiae* with Ca^2+^ ions, the FTIR analysis was performed. According to Ojeda *et al*.^25^ the FTIR analysis is an inexpensive and rapid tool to determine the chemical properties of microbial cells and also to identify the surfaces functional groups of microbes.

In order to eliminate the possibility that the observed changes on the FT-IR spectra result only from different pH condition, the FT-IR spectra of yeast at different pH without calcium ions were recorded. Supplementary Fig. S3 presents the FT-IR spectra of incubated *S*. *cerevisiae* at different pH without the calcium solution. These results indicate that a change only in the pH of the cells environment did not significantly affect the resulting FT-IR spectrum.

**Figure 2:**
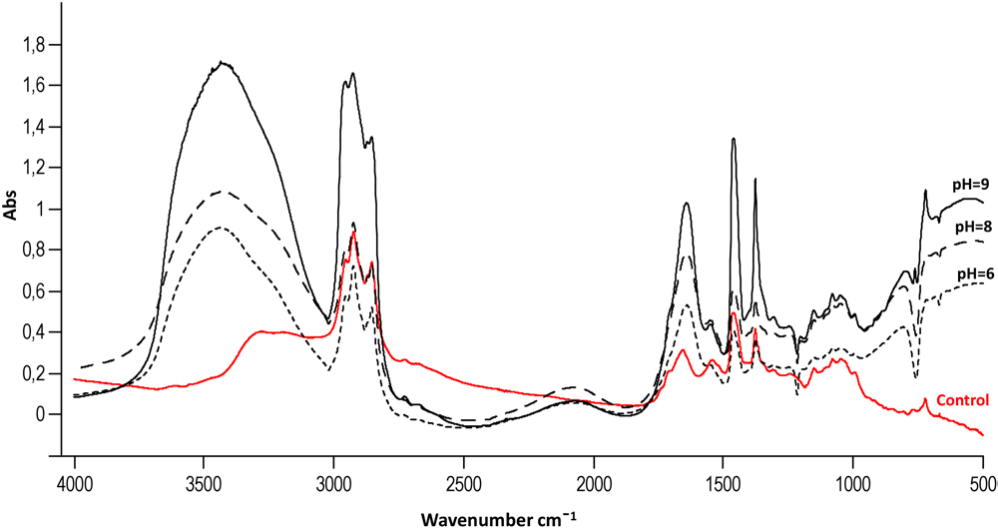
The FTIR spectra of unmodified and modified *S*. *cerevisiae* pellets at pH 6, 8 and 9.

Figure 2 shows the FTIR spectra of *S*. *cerevisiae* unmodified and modified by calcium ions at different pH. Comparing the spectra of the unmodified and modified yeast, many important changes can be observed. After the surface modification, a new signal at v = 722 cm^−1^ appeared - it may derive from P = O vibrations from phosphate/hydrogen phosphate groups. There is also a shift of signal from v = 1652 cm^−1^ to v = 1644 cm^−1^ which is associated with the vibrations of the carboxyl and amino group^17,19,26^. These changes can indicate the creation of bonds between the calcium cation and particular functional groups responsible for this vibration. Moreover, as a result of the modification, merging signals at v = 1715 cm^−1^ and v = 1460 cm^−1^ present in the spectrum of unmodified yeast can be observed. Another characteristic band appears at v = 1377 cm^−1^ and derives from the stretching vibration of C-N from surface proteins. These changes are probably related to the complexation reactions between the calcium cation and O- and N- donors of proper functional group^26,27^. The obtained data prove that the *S*. *cerevisiae* exhibits the ability of the sorption of divalent metal cations such as calcium ions^27^. Furthermore, it can be observed that the most significant changes occur in the spectrum of the yeast modified by 5 mM Ca(NO_3_)_2_ solution at pH = 9, which is caused by the total deprotonation of -COO^−^, -OPO_3_^2−^ and -NH_2_ groups. These results are also confirmed by the zeta potential measurement in this study - the highest dispersion stability of the system was observed at the pH range from 8 to 10^21^. Therefore, under these conditions, the tested (bio)colloid is characterized by a large active surface and the highest availability of functional groups able to bind calcium ions. This indicates that the most effective biosorption process occurs in these conditions.

### The impact of the S. cerevisiae surface functional groups modification on their electrophoretic separation at different pH

**Figure 3:**
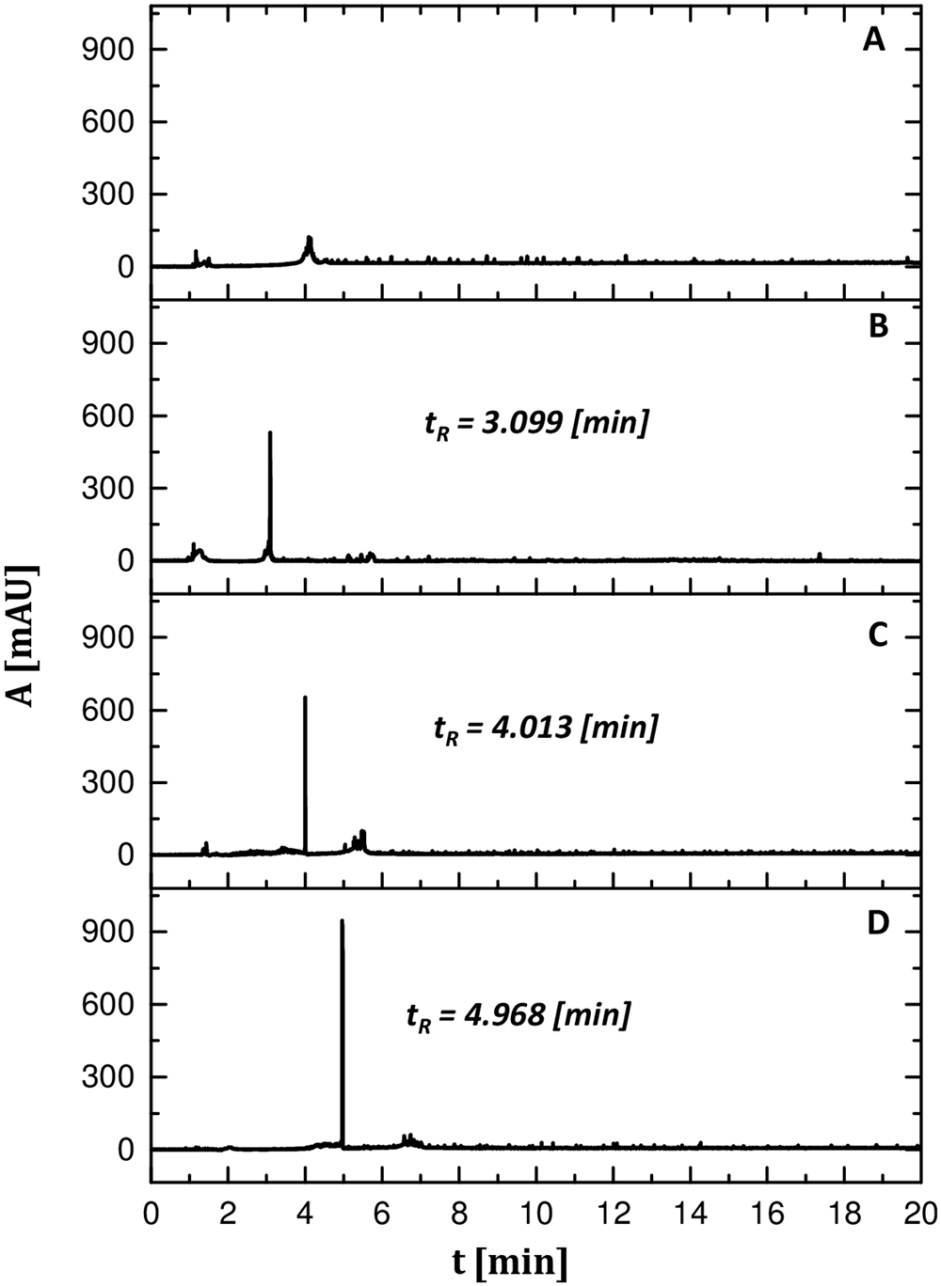
Electropherogram of non-modified *S*. *cerevisiae* (**A**); modified *S*. *cerevisiae* by 5 mM Ca(NO_3_)_2_ at pH = 6 (**B**), pH = 8 (**C**) and pH = 9 (**D**). Conditions: inlet buffer: TBH (pH = 7.31), outlet buffer: TB (pH = 7.98), suspensive buffer: TB (pH = 7.98); I = 100 μA, U = 15 kV, t = 23 °C, λ = 214 nm, L = 33.5 cm, L_eff_ = 25 cm, ϕ = 100 μm, injection: 20 mbar t = 8 s.

The chosen electrolyte for the modification of the yeast surface was Ca(NO_3_)_2_ at the concentration of 5 mM. According to Dziubakiewicz *et al*. the type and concentration of the background electrolyte have a significant influence on the cells viability^20^. The number of viable cells increased along with the decrease in the electrolyte ionic strength: 0.1 M > 0.01 M > 0.005 M. To determine the effect of the surface modification of *S*. *cerevisiae* on the electrophoretic mobility, the electrophoretic analysis was conducted. Figure 3 shows the electropherograms of the yeast unmodified and modified by calcium ions at different pH conditions. The electromigration time of the yeast modified by the calcium solution of pH 6, 8 and 9 was 3.099 (RSD = 4.59%), 4.013 (RSD = 4.73%) and 4.099 (RSD = 3.03%) min respectively.

The results indicate that the surface modification of (bio)colloid has a significant impact on its electrophoretic mobility. This phenomenon was also observed by Pomastowski *et al*.^28^ in the case of bacteria. They stated that the change in the electrophoretic mobility of bacteria cells and the reduction of repulsive forces have resulted in clumping of cells and a signal amplification. Moreover, after the modification, the sharpening of the peaks and improvement of the shape of the base line on the electropherogram as well as the reduction in the number of aggregates and the improvement of the reproducibility can be observed; it was evidenced by a significant reduction in the relative standard deviation value in comparison to unmodified yeast. Another interesting observation is the effect of pH on modified (bio)colloid behaviour during the electrophoretic analysis. With the increase in the pH of medium in which sorption was conducted, the increase in the electromigration time of modified yeast cells and as a decrease in the electrophoretic mobility (38.1 ± 2, 27.8 ± 1 and 24.5 ± 1 [10^−5^ cm^2^Vs] for pH 6, 8 and 9, respectively) were observed (Fig. 3). However, these effects were not observed for the yeast cells incubated at different pH but without the presence of calcium ions, as demonstrated in Supplementary Fig. S4.

The highest reproducibility (RSD < 5%) of the electrophoretic analysis was obtained for *S*. *cerevisiae* which was modified with Ca(NO_3_)_2_ solution at pH = 9. This phenomenon is probably connected with the total deprotonation of surface functional groups, which is also confirmed by the results of the spectroscopic and potentiometric analysis. The total deprotonation of active functional groups at pH = 9 causes the high dispersion stability (zeta potential measurements) and high availability of ion binding sites at the starting point of the sample preparation stage, which determines the creation of stable electrokinetic clumping during the yeast cells electroanalysis. A high availability of the deprotonated functional groups on the surface of *S*. *cerevisiae* allows binding of a large amount of calcium ions which promote the formation of aggregate systems.

Moreover, the study of the size of modified and non-modified yeast cells at different pH **(**Supplementary Fig. S5**)** indicates that the change occurring only in the pH of the incubation does not have a significant impact on the size distribution of the examined cells, while the modification of cells by calcium causes the unification of their size. This phenomenon may also contribute to the improvement of their electrophoretic separation.

Microscopic and spectrometric studies of modified and non-modified yeasts were performed in order to highlight the changes in the molecular profile of (bio)colloids under different pH conditions.

The MALDI-TOF MS spectra of yeast incubated at different pH (6, 8 and 9) without calcium ions are present in Supplementary Fig. S6. As it can be observed the incubation of *S*. *cerevisiae* at different pH does not significantly affect their molecular profile in comparison to the control - *S*. *cerevisiae* sample taken directly from growth media. On the obtained spectra only slight changes can be observed. What may be observed is the appearance of signals at about 2776 and 5853 m/z as well as the disappearance of signals at 8128 m/z on the spectra of yeast at different pH in comparison to the control sample. Moreover, it can be noticed that the spectra of the yeast incubated at pH 9 are the most similar to control. There are a number of signals which are not present on the spectra of the yeast incubated at pH 6 and 8 e.g. at 4451, 5178, 7101 and 9433 m/z. This result suggests that the cells incubation at pH 9 causes the smallest changes in the *S*. *cerevisiae* molecular profile.

**Figure 4:**
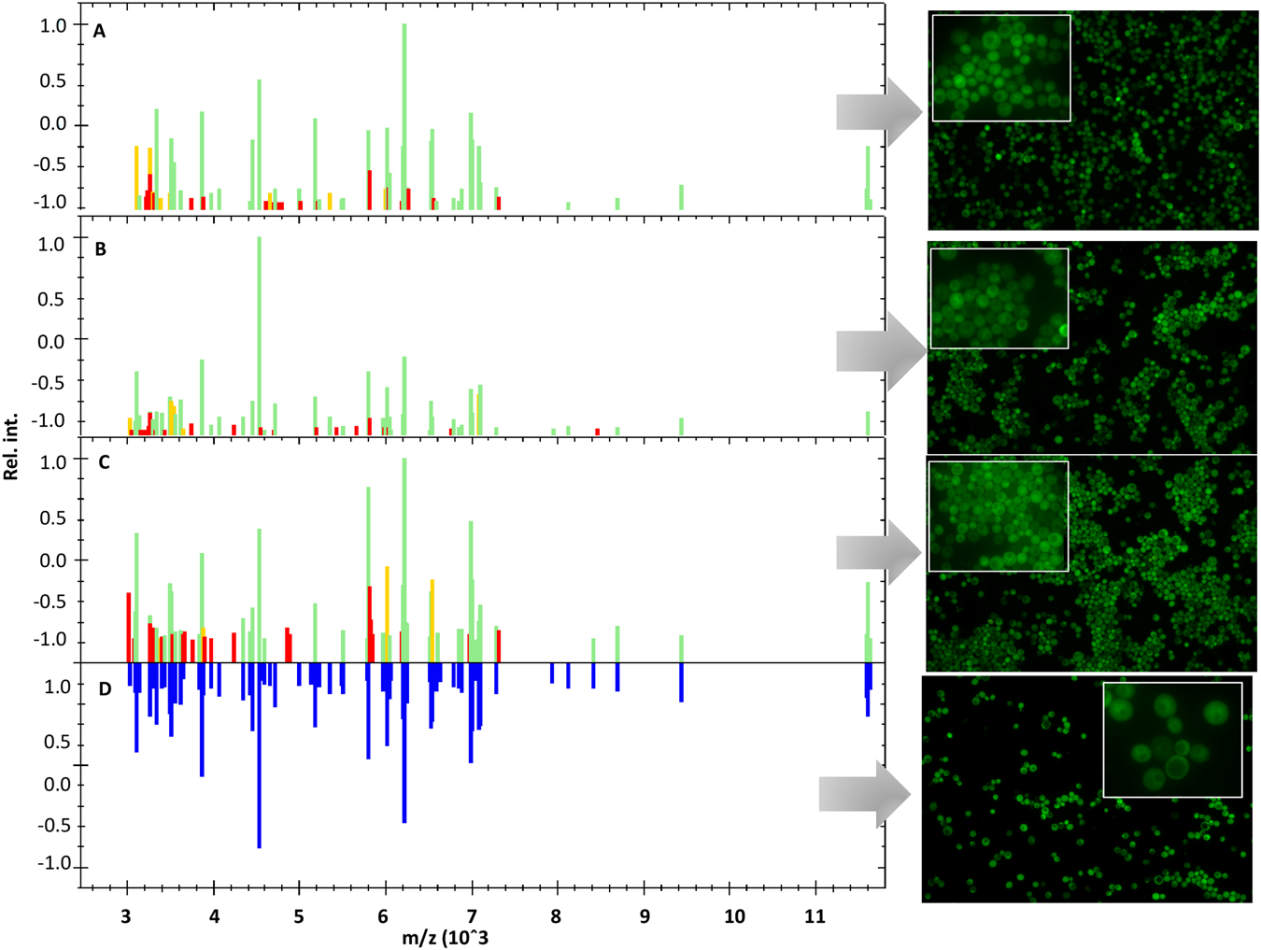
MALDI-TOF MS spectra and microscopic images of *S*. *cerevisiae* suspended in 5 mM Ca(NO_3_)_2_ solution at pH = 6 (**A**), pH = 8 (**B**), pH = 9 (**C**) and native *S*. *cerevisiae* (**D**), where green signals are correlated with control sample, yellow signals are shifted and red signals are disappeared in compare with native sample.

Figure 4 shows the MALDI spectra and microscopic images of *S*. *cerevisiae* suspended in calcium solution at pH 6, 8 and 9 and Table 1 presents signals that appear and disappear in spectra for individual samples compared to the control sample.

**Table 1:** Summarizes of signals observed on MALDI-TOF MS spectra (Fig. 4), where (−) mean disappearance of signal in comparison to control and (+) mean appearance of signal in comparison to control.

pH 6		pH 8		pH 9	
(−)	(+)	(−)	(+)	(−)	(+)
3023	3213	3424	3041	3023	3007
3097	3228	3834	3159	3144	3080
3424	3238	3866	3203	3296	3268
3550	3262	3876	3239	3543	3279
3643	3266	4421	3268	3643	3291
3834	3307	4570	3322	3863	3318
3863	3734	4654	3430	3972	3400
3876	3884	4987	3735	4064	3522
4348	4607	5137	3863	4523	3646
4660	4698	5230	4236	4570	3661
4691	4758	5483	4550	4654	3762
5137	4792	5781	4702	4716	3897
5781	5004	6234	5188	4987	3969
5969	5209	6249	5432	5137	4232
6064	5821	6596	5657	5230	4856
6509	6031	6636	5793	5352	4881
6636	6042	7041	5816	5483	5812
7041	6188	8418	5982	5995	5829
7942	6269	11588	6025	6064	5840
8418	6554	11637	6752	6636	6185
	7319		8460	6785	6972
				7942	6974
				8125	7320

**Figure 5:**
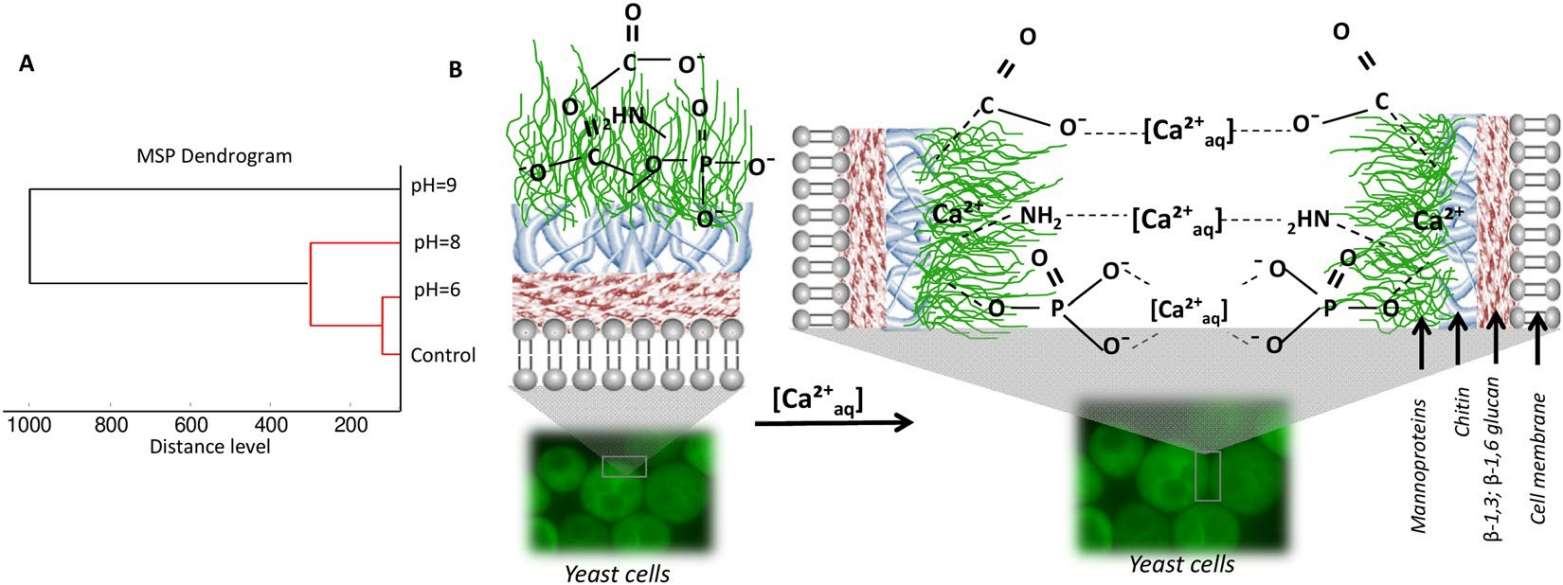
MSP dendrogram for MALDI-TOF MS spectra of *S*. *cerevisiae* modified with 5 mM Ca(NO_3_)_2_ solution at pH 6, 8, 9 and native *S*. *cerevisiae* (non-modified) (**A**). Model of yeast cell wall controlled clumping mechanism (**B**).

The MALDI spectra of native yeast cells and yeast incubated with calcium solution at different pH show obvious changes. On the MALDI spectra of yeast modified by calcium at pH 6, the new signals e.g. at 3213, 5004 and 7319 m/z were registered. Moreover, in comparison with the control, some signals e.g. m/z = 3023, 5781 and 8418 disappeared or move to higher m/z values **(**Fig. 4A**)**. The same phenomenon can be observed in case of yeast modified by calcium ions at pH 8 and 9 **(**Table 1**)**. However, the largest changes in the molecular profile of *S*. *cerevisiae* were observed after its incubation in Ca(NO_3_)_2_ at pH 9 **(**Fig. 4C**)**. It can also be observed that the modification with calcium ions leads to the disappearance of the same signals regardless of the incubation pH value e.g. at 5137 and 6636 m/z. Moreover, the same signals disappeared on the spectra of yeast modified in lower values of pH (6 and 8) (e.g. at 3424, 3834 and 3876 m/z) as on the spectra of native yeast. A similar relationship can be seen for the spectra at pH 8 and 9. The some signals present on the native yeast spectra disappear (e.g. at 4570, 4987 and 5483 m/z) in this condition and one signal not present on the control spectra has appeared (3268 m/z). The cluster analysis of MSP (Main Spectrum Profile) spectra of control and yeasts incubated in the calcium solution at pH = 6, 8 and 9 shows the significant differences between samples **(**Fig. 5A**)**. The largest number of similarities were observed for a native sample and yeast incubated in Ca(NO_3_)_2_ solution at pH = 6. The greatest change in molecular profiles, as compared to the control sample, was observed for the yeast incubated in calcium solution at pH = 9.

The spectrometric study shows the differences in the MALDI spectra of the incubated yeast cells in calcium solutions in comparison with the control sample (unmodified yeast). The changes were observed at the molecular level of ribosomal, cytosol and membrane proteins. It is known that the presence of the Ca^2+^ ions in the culture medium may strongly influence the expression of proteins in *S*. *cerevisiae* cells, mainly ribosomal or ribosome-associated proteins as well as proteins related to the cell wall. High concentrations of Ca^2+^ leads to the overexpression of cell wall integrity regulators as a response to the osmotic and ionic changes that occur in the media^29^ which can explain the changes of protein profiles observed in this work during MALDI TOF/MS analysis. Observed signals at specific m/z value can be assigned to presence of specific proteins occurring in yeast cells. According to the UniProt database, the disappearance of signals at 6636 m/z after the yeast incubation with calcium ions at pH 6, 8 and 9 is probably associated with the decrease in the expression level of H/ACA ribonucleoprotein complex subunit 3 which occurs in nucleus and is responsible for the RNA binding process. In turn, the disappearance of signal at 7041 m/z on the yeast spectra incubated with Ca^2+^ at pH 6 and 8 indicates the cessation of the production of one of the mitochondrial proteins; in the same condition the appearance of signals at 5821 and 3203 m/z derivating from cytochrome protein was noted. Moreover, many changes on each spectra of modified yeast in comparison to spectra of native yeast may be observed, which indicates a change in the expression of membrane proteins. There can also be seen a disappearance of signals at 6509, 6249 and 3643 m/z at pH 6, 8 and 9, respectively as well as the appearance of new signals at 6752 m/z at pH 8 and 6974 m/z at pH 9.

The MALDI-TOF MS technique is a new approach enabling for a quick and accurate identification of microorganisms. However, this method requires a relatively high concentration of the microbial sample. According to Pomastowski *et al*.^28^, less than 10^4^ cells per spot is not enough to generate the spectrum and the optimal number of cells amounts 10^5^–10^9^ per spot. For this reason, an extremely important aspect is the development of an effective method of the concentration of microbiological samples so that it does not affect matching of the obtained spectrum with the reference spectra of the microorganisms deposited in the database. The precontamination method of modifying microbial cells by calcium ions by the capillary electrophoresis presented in this work may be considered an analytical solution of this problem. Obtained results indicate that the modification of yeast cells by calcium ions at strictly defined pH leads to its effective electrofocusing by the capillary electrophoresis. Moreover, this sample modification does not affect the identification degree. The score for modified and non-modified yeast was about 2 (using BioTyper database), which ensures unambiguous identification.

The microscopic observation shows that the highest aggregation of the yeast cells was achieved for the system, where solution at pH = 9 was used. In the case of the calcium solution at pH = 6, cluster formation wasn’t observed **(**Fig. 4A**)**. Moreover, microscopic results prove that the yeast surface changes with a calcium ion affect the aggregates formation. A similar phenomenon was observed by Dziubakiewicz *et al*. for the bacterial cells^6^. As a result of the bacteria surface charge modification by calcium ions, the cells created a compact aggregates, whereby, fewer high-intensity signals on the electropherograms were observed. They attributed this score to the bridging effect of calcium ions between bacterial cells. According to the well-known theory of dicationic bridges, carboxyl and amine surface groups are involved in the formation of aggregates. Partially deprotonated carboxyl groups and protonated amino groups are not able to form bridges between (bio)colloids. After yeast centrifugation from calcium salt solution and suspended in TB buffer, probably borate ions associate and/or complex adsorbed calcium ions. The created ions of TB buffer respectively associate components of the microorganisms surface. In turn, yeast suspended in a calcium solution at pH = 8 form aggregates with part-compact structure **(**Fig. 4B**)**.

Deprotonated carboxyl groups allow for the creation of a cationic bridge between the surfaces of the yeast cell wall **(**Fig. 5B**)**. After suspending the system in TB buffer, the metal cations previously adsorbed on the (bio)colloids surface, are associated and/or complexed by suitable buffer ions. In turn, yeast suspended in the calcium nitrate solution at pH = 9 forms aggregates of compact structure **(**Fig. 4C**)**.

Deprotonated carboxyl surface groups allow for the creation of a series of the cationic bridges between (bio)colloids surfaces **(**Fig. 5B**)**. After transfering the system to the TB buffer, the metal cations previously adsorbed on the (bio)colloid surface are then associated and/or complexed by suitable buffer ions. Based on the above considerations, it can be seen that with the increasing pH the buffer growth of (bio)colloid particles weight (cells clumping) is observed. Therefore, when the size and masses of (bio)colloids are increasing, the electrophoretic mobility of (bio)colloids is decreasing.

## Conclusion

Capillary zone electrophoresis allows for the determination of a variety of biological systems such as bacteria, yeast and fungi. However, the complexity of the microorganism surface morphology forced us to carry out a series of research on the physicochemical properties in order to interpret the phenomena occurring at the interface of (bio)colloids. The characterization of microorganisms surface functional groups and its modification allowed us to develop a effective method for the electrofocusing of yeast cells. The use of potentiometric titration and the FTIR spectroscopic analysis allowed for the identification and determination of an amount of dominant surface functional groups of yeast, namely carboxyl (16%), amino/hydroxyl (16%) and phosphate/ hydrogen phosphate (68%) groups. The measurement of surface charge was another step to broaden the knowledge of *S*. *cerevisiae* electrochemistry. The knowledge of the surface charge and dominant surface functional groups of *S*. *cerevisiae* led to the selection of appropriate analysis conditions in order to eliminate the adverse effects of yeast adhesion to the capillary wall and uncontrolled clumping of native species. For this purpose, yeast surface was modified with calcium salt solution at different pH. Moreover, microscopic, spectrometric and spectroscopic studies performed for non-modified and modified *S*. *cerevisiae* have shown that under specified conditions the tested system tends to aggregate. This phenomenon explains the stability of (bio)colloid subjected to the biosorption process in electrophoretic separation conditions. In addition, it was observed that along with the increasing pH of modifier the weight and surface of particles of colloidal system increases, which results in the reduction of the (bio)colloid electrophoretic mobility. Obtained results indicate that the proposed new sample preparation approach of wild microorganisms strains may be a foundation for the application of capillary electrophoresis in diagnostics labolatory in the future. Moreover, the results show that the different pH and modification of the cells by calcium ions influence the molecular profiles of yeast cells but do not affect the identification quality by the MALDI-TOF MS equipment with the BioTyper database. Such results may provide a basis enabling for coupling of capillary electrophoresis and the MALDI-TOF MS analysis.

## Materials and Methods

### Materials

All solvents and materials were purchased from Avantor (Gliwice, Poland) and Sigma Aldrich (Poznan, Poland). Ultra-pure water was purified using the Milli-Q RG system (Millipore Intertech, Bedford, MA, USA).

### Culturing and identification of Saccharomyces cerevisiae cells isolated from baker’s yeast

Pure culture of *Saccharomyces cerevisiae* cells was obtained from baker’s yeast (Gliwice, Poland) by a serial dilution method using a sterile saline solution (0.87%). 100 µl of 10^−5^ dilution of yeasts was smeared on the YPD (Yeast Peptone Dextrose) agar plates (Sigma Aldrich, Poznan, Poland) and incubated for 24 h at 37 ± 0.5 °C. Subsequently, single colonies were transferred on the YPD slants, incubated overnight at 37 ± 0.5 °C and stored at 4 ± 0.5 °C. In the experiment overnight cultures of yeast cells were transferred into the YPD Broth (Sigma Aldrich, Poland) and cultivated for 24 h at 37 ± 0.5 °C.

In order to identify the *S*. *cerevisiae* strain used in this work, the DNA isolation from yeasts cultures using Extractme^®^ DNA Yeast Kit (BLIRT S.A., Gdansk, Poland) was performed, which was followed by the amplification of two genome regions by PCR reaction – (1) 28 S using 28S_NL1: 5-GCATATCAATAAGCGGAGGAAAAG-3 and 28S_NL4: 5-GGTCCGTGTTTCAAGACGG-3 primers as well as (2) internal transcribed spacer using ITS1: 5-TCCGTAGGTGAACCTGCGG-3 and ITS4: 5-TCCTCCGCTTATTGATATGC-3 primers (Laboratory of DNA Sequencing and Oligonucleotide Synthesis, Institute of Biochemistry and Biophysics, Polish Academy of Sciences, Warsaw, Poland). For the amplification, thermostable Taq DNA polymerase (Qiagen, Hilden, Germany) and Mastercycler^®^ pro S thermocycler (Eppendorf AG, Hamburg, Germany) were employed. PCR steps were as follows: 1. initialization: 95 °C, 3 min.; 2. denaturation: 95 °C, 15 s; 3. annealing: 53 °C, 15 s; 4. elongation: 72 °C, 40 s (steps 2. – 4. repeated 30×); 5. final elongation: 72 °C, 2 min.; 6. final hold: 10 °C. Finally, the direct sequencing of PCR products via sanger dideoxy sequencing technology was performed. Obtained consensus sequences were compared with the known 28 S and ITS genes at The National Center for Biotechnology Information (NCBI) BLAST database^30^. The evolutionary tree of the identified yeast strain was inferred based on the Neighbor-Joining method (bootstrap analysis from 500 replicates, values <50% not shown) using the MEGA7 software^31^. As an alternative method of yeast identification, the MALDI TOF MS, performed according to previously described procedure by Railean-Plugaru *et al*.^32^ was used. The recorded mass spectra were processed using the MALDI Biotyper Systems (Bruker Daltonics, Germany) chosen for routine identification in clinical microbiology laboratories worldwide.

### Determination of S. cerevisiae viability at different pH

Yeast were suspended in water adjusted to desired pH (3–12) by addition of 0.1 mM HCl or 0.1 mM NaOH and incubated for 60 min at 37 °C. After this time the yeast sample was twice washed with sterile water. To each sample methylene blue was added. *S*. *cerevisiae* cells viability was determined using the fluorescence microscope Zeiss Axiocom D1 (Germany) with Axio Vision 4.8. software and set of filters 43 He and 38.

### Zeta potential measurements

The 50 mg of yeast pellet were transferred to 6 mL of NaNO_3_ (5 mM) at pH range 2–11. The sample was vortexed (21 ± 0.5 °C, 1 min) and sonicated at 25 ± 0.5 °C (2 min). The zeta potential measurements of yeast were performed by Zetasizer Nano Series (Malvern Instruments, Malvern, UK). The value of the zeta potential was calculated according to Smoluchowski equation:

1 $$\zeta =\,\frac{{\mu }_{e}\cdot \eta }{\varepsilon }$$

where μ_e_ stands for electrophoretic mobility, η is medium viscosity and ε is dielectric constant.

### Potentiometric titration

50 mg of yeast cells was transferred to 10 mL of 5% glucose solution. After 8 minutes the yeast suspension was centrifuged (21 ± 0.5 °C, 9000 rpm, 15 min). The supernatant was transferred into a glass vessel and the yeast precipitate was suspended in 5 mL of NaCl (10 mM). Before the analysis, CO_2_ was removed by nitrogen flow. The precipitate and supernatant were sequentially titrated with HCl (0.1 M) to pH 3 (Cerko Lab System, Gdansk, Poland with PT1000 probe and ERH-13 electrode with Cell software). Another sample prepared in the same way was titrated with NaOH (0.1 M) to pH 11. pKa values were calculated by Protofit 2.1rev1 free software licensed under the Gnu General Public License (GPL)^33^.

### Preparation of yeast cells modified by calcium ions at different pH and yeast cells at different pH

*S*. *cerevisiae* samples were suspended in 5 mM Ca(NO_3_)_2_ adjusted to the desired pH (6, 8 and 9) by the addition of saturated Ca(OH)_2_. After 60 minutes of incubation (20 ± 0.5 °C), the suspension was centrifuged (21 ± 0.5 °C, 9000 rpm, 15 min). The resulting precipitate was washed twice with water to remove unbound calcium ions. The same procedure was used to obtain the control sample of yeast at different pH, but instead 5 mM Ca(NO_3_)_2_, water adjusted to desired pH using 0.1 mM NaOH solution was used. As for the control sample, yeast taken directly from media were used.

### Fourier transform infrared spectroscopy (FTIR) spectroscopic analysis

For modified and non-modified *S*. *cerevisiae* pellets and Ca(NO_3_)_2_ salt, FTIR spectra at range ν = 400–4000 cm^−1^ using the KBr methods (FT-IR Spectrum 2000, Perkin-Elmer, Waltham, USA) were registered. Non-modified *S*. *cerevisiae* pellets and Ca(NO_3_)_2_ salt were considered as controls.

### Spectrometric and microscopic analysis and determination of yeast cell size

In case of the microscopic analysis, the modified and non-modified *S*. *cerevisiae* samples were stained with acridine orange (0.12 μg/mL) for 5 min in the dark, then centrifuged at 4000 rpm (5 min). The supernatant was eliminated to remove the unbound dyes and yeast cells were detected through the green fluorescence using the Zeiss Axiocom D1 fluorescence microscope. For the spectrometric analysis the sample of modified and non-modified *S*. *cerevisiae* was analysed according to the previously described methodology^28^. The size of non-modified and modified *S*. *cerevisiae* cells at different pH was determined using the Mastersizer 3000 (Malvern).

### Capillary zone electrophoresis analysis

50 mg of modified and non-modified *S*. *cerevisiae* were transferred to the outlet TB buffer tris(hydroxymethyl)aminomethane (TRIS) C_TRIS_ = 4.5 mM, boric acid (BA) C_BA_ = 50 mM; pH = 7.98) as the inlet buffer TBH (TRIS C_TRIS_ = 4.5 mM, BA C_BA_ = 50 mM, hydrochloric acid C_HCl_ = 3.31 mM; pH = 7.31) was used. Directly before the CZE analysis the obtained yeast sample was placed in an ultrasonic bath (2 min) (Ultrasonic Cleaner USC600THD, Radnor, Pennsylvania). Before the electroanalysis of yeast the EOF (t_d_ = 1.23 min) was determined by thiourea injection using the pressure mode (10 mbar, 10 s).

The CZE analysis was performed using PA 800 plus (Beckman Coutner system, Brea, CA, USA) equipped with a DAD detector with the use of fused silica capillaries (id = 75 μm; L_tot_ = 33.5 cm; L_eff_ = 25 cm; Composite Metal Services, Shipley, UK). The yeast samples were injected in the capillary using a pressure mode (20 mbar, 8 s). The analysis were performed at a constant voltage = 15 kV and at the temperature 23 ± 0.5 °C. The samples were detected at λ = 214 nm. The CZE analysis was performed in *pseudo-isotachophoretic* mode^6^.

## Electronic supplementary material

Supplementary information
