# Supplementary material for: The influence of different pH on the electrophoretic behaviour of Saccharomyces cerevisiae modified by calcium ions
Source: Sci Rep. 2018 May 8;8:7261. doi: 10.1038/s41598-018-25024-4 (PMC5940755; doi:10.1038/s41598-018-25024-4)
Supplement: Supplementary file 1 — Supplementary information [file 41598_2018_25024_MOESM1_ESM.pdf]

Supplementary Information

The influence of different pH on the electrophoretic behaviour of *Saccharomyces cerevisiae* modified by calcium ions

Agnieszka Rogowska[1,2], Paweł Pomastowski[1], Michał Złoch[2], Viorica Railean-Plugaru[1], Anna Król[1,2], Katarzyna Rafińska[1,2], Małgorzata Szultka-Młyńska[2], Bogusław Buszewski[1,2]*

[1]Centre for Modern Interdisciplinary Technologies Nicolaus Copernicus University, Wileńska 4, 87-100 Torun, Poland

[2]Department of Environmental Chemistry and Bioanalytics, Faculty of Chemistry, Nicolaus Copernicus University, Gagarina 7, 87-100 Torun, Poland, email: bbusz@chem.uni.torun.pl

** Corresponding author:*

Prof. Dr. Bogusław Buszewski

E-mail: bbusz@chem.umk.pl,

Ph. +48 56 611 43 08,

pH	Live [%]	Dead [%]
**3**	83 ± 3	17 ± 3
**4**	91 ± 6	9 ± 6
**5**	93 ± 6	7 ± 6
**6**	92 ± 1	8 ± 1
**7**	87 ± 8	13 ± 8
**8**	90 ± 6	10 ± 6
**9**	94 ± 3	6 ± 3
**10**	83 ± 8	17 ± 8
**11**	87 ± 4	17 ± 8
**12**	73 ± 5	27 ± 5

Supplementary Table S1. The percentage of live and dead yeast cells after 30 min of its incubation at different pH.

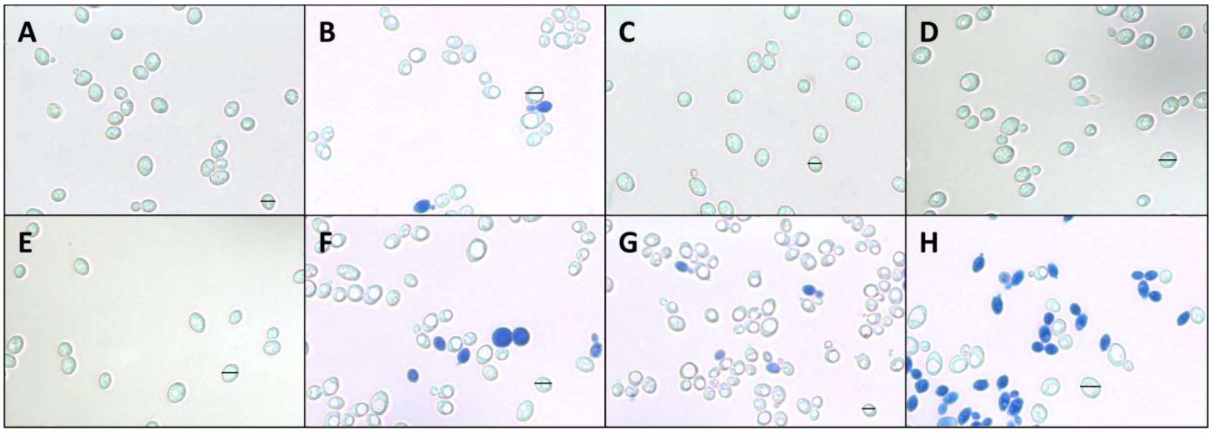

Supplementary Figure S2. Microscopic image of yeast cells after its incubation in water (A) and at pH 3 (B), 6 (C), 8 (D), 9 (E), 10 (F), 11 (G) and 12 (H) (bar 5 μm).

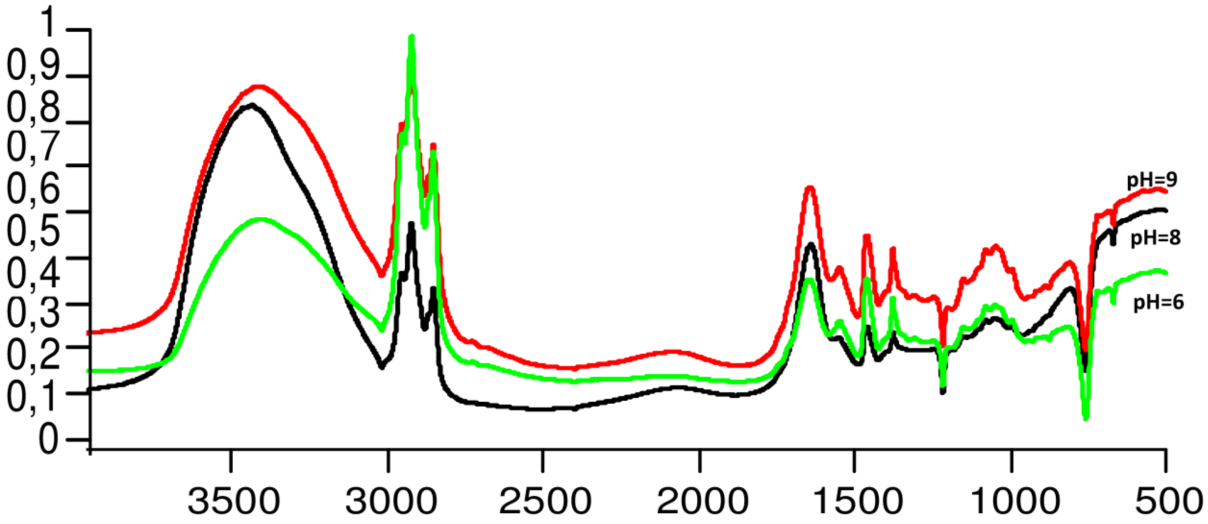

Supplementary Figure S3. The FTIR spectra of *S. cerevisiae* pellets without calcium ions at pH 6, 8 and 9.

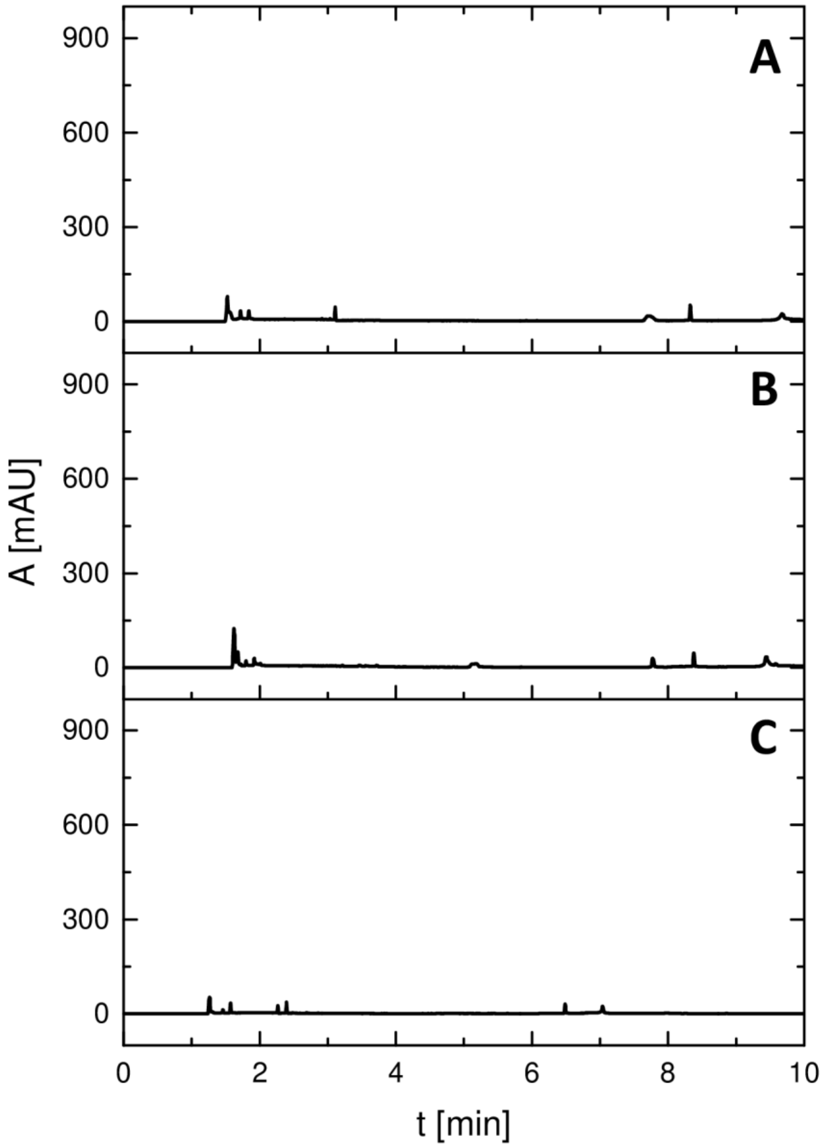

Supplementary Figure S4. Electropherogram of *S. cerevisiae* incubated at pH = 6 (A), pH = 8 (B) and pH = 9 (C). Conditions: inlet buffer: TBH (pH = 7.31), outlet buffer: TB (pH = 7.98), suspensive buffer: TB (pH = 7.98); I=100μA, U=15kV, t=23°C, λ=214 nm, L=33.5 cm, L_{eff}=25 cm, ϕ=100 μm, injection: 20 mbar t = 8 s.

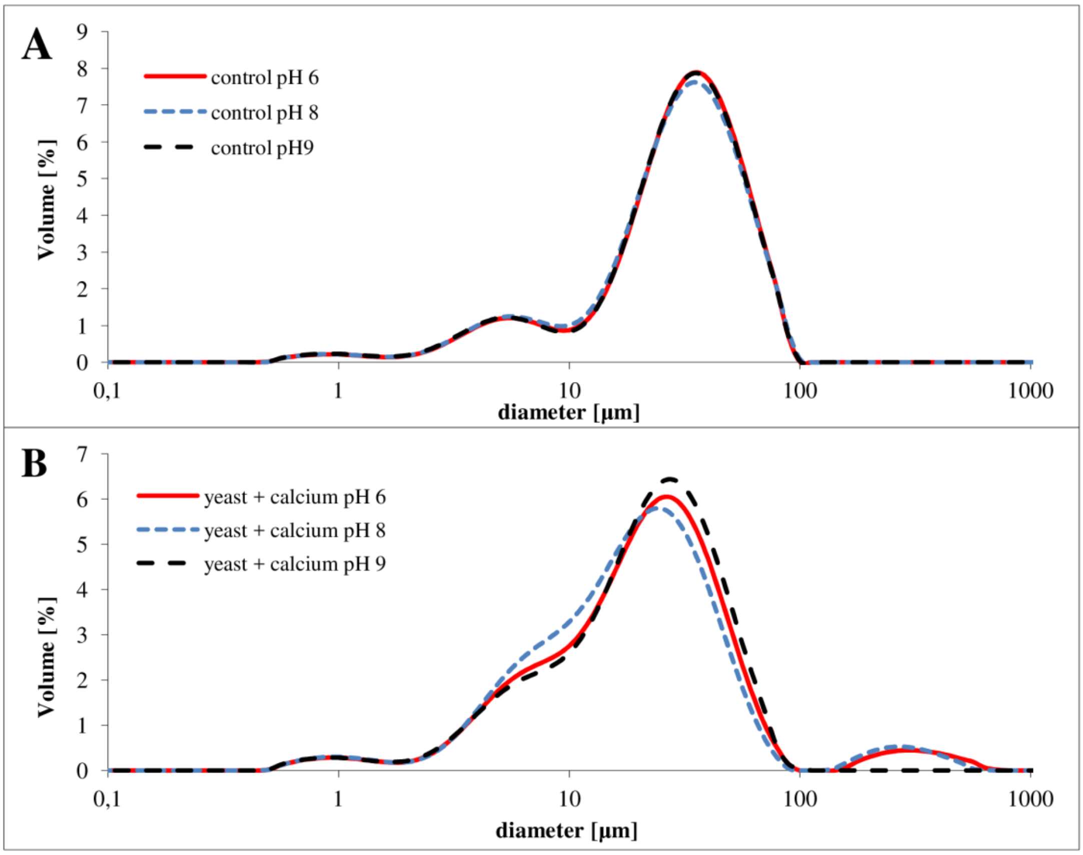

Supplementary Figure S5. The size distribution of yeast cells (A) after its incubation at different pH (6, 8 and 9) and (B) after its incubation in $Ca(NO_3)_2$ solution at different pH (6, 8 and 9).

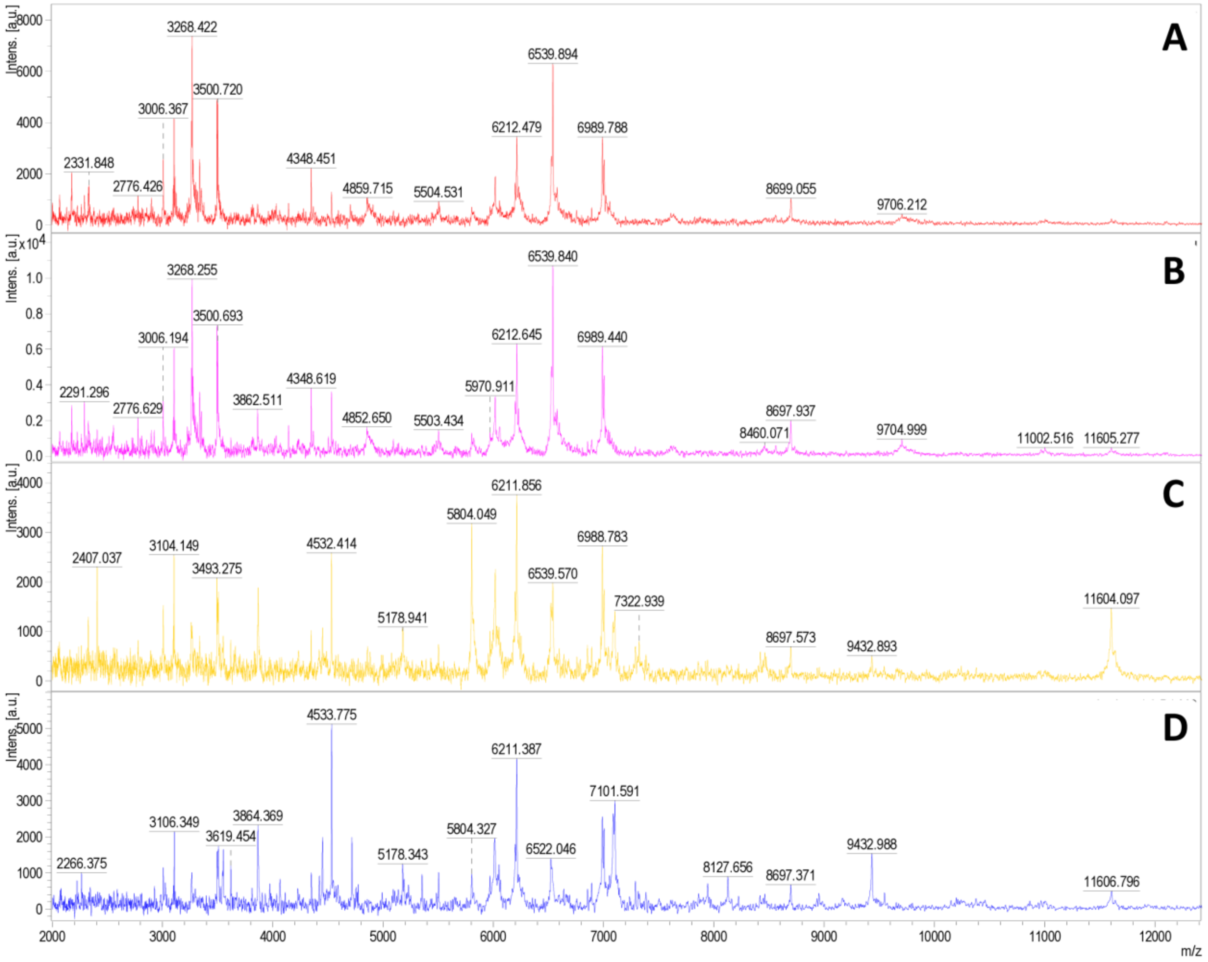

Supplementary Figure S6. MALDI-TOF MS spectra of *S. cerevisiae* incubated at pH 6 (A), 8 (B), 9 (C) and native yeast cells (D).
